# Profile of upregulated inflammatory proteins in sera of Myasthenia Gravis patients

**DOI:** 10.1038/srep39716

**Published:** 2017-01-03

**Authors:** Carl Johan Molin, Elisabet Westerberg, Anna Rostedt Punga

**Affiliations:** 1 Uppsala University, Department of Neuroscience, Clinical Neurophysiology, BMC, Husargatan 3, 75237 Uppsala, Sweden

## Abstract

This study describes specific patterns of elevated inflammatory proteins in clinical subtypes of myasthenia gravis (MG) patients. MG is a chronic, autoimmune neuromuscular disease with antibodies most commonly targeting the acetylcholine receptors (AChRab), which causes fluctuating skeletal muscle fatigue. MG pathophysiology includes a strong component of inflammation, and a large proportion of patients with early onset MG additionally present thymus hyperplasia. Due to the fluctuating nature and heterogeneity of the disease, there is a great need for objective biomarkers as well as novel potential inflammatory targets. We examined the sera of 45 MG patients (40 AChRab seropositive and 5 AChRab seronegative), investigating 92 proteins associated with inflammation. Eleven of the analysed proteins were significantly elevated compared to healthy controls, out of which the three most significant were: matrix metalloproteinase 10 (MMP-10; p = 0.0004), transforming growth factor alpha (TGF-α; p = 0.0017) and extracellular newly identified receptor for advanced glycation end-products binding protein (EN-RAGE) (also known as protein S100-A12; p = 0.0054). Further, levels of MMP-10, C-X-C motif ligand 1 (CXCL1) and brain derived neurotrophic factor (BDNF) differed between early and late onset MG. These novel targets provide valuable additional insight into the systemic inflammatory response in MG.

Myasthenia gravis (MG) is a chronic autoimmune disease, which is caused by antibodies against receptors at the neuromuscular junction. Disruption of neuromuscular transmission results in symptoms of fatigue in proximal skeletal muscles; predominantly facial, bulbar and limb muscles^1^,^2^. In approximately 85% of the patients, IgG1 antibodies are directed towards the nicotinic acetylcholine receptors (AChR+)^3^, while a smaller portion of MG patients possess IgG4 antibodies towards muscle specific tyrosine kinase (MuSK+)^4^ or low density lipoprotein receptor-related protein 4 (Lrp4)^5^. Patients who do not have detectable serum antibodies are termed seronegative. The antibody subtype is important to consider, since both symptoms and treatment response substantially differ between the different serological MG subgroups^6^. MG can be further subdivided into early onset (EOMG) or late onset (LOMG), depending on the age at which the patient first develops myasthenic symptoms. Although there is no absolute consensus at which age to draw the line between EOMG and LOMG^7^, the most commonly applied cut-off point for LOMG is 50 years of age. The distinction between EOMG and LOMG is important, since patients with LOMG may have a different disease course and typically do not present thymic hyperplasia, which is frequently seen in EOMG^8^.

As of yet, there are no circulating serum biomarkers that correlate with the disease state between patients or different MG subtypes^9^. The levels of AChR or MuSK antibodies generally do not correlate well with MG disease severity, neither between patients or inter-individually. Furthermore, clinical muscle fatigue may not be an entirely reliable parameter to follow in clinical trials due to expected fluctuations from one day to another, and even during the course of one day. Recently, microRNA (miRNA) have been described as potential biomarkers regarding miR-150-5p and miR-21-5p for AChR + MG^10^,^11^ and the let7 family for MuSK + patients^12^. Both miR-150-5p and miR-21-5p are involved in the regulation of the autoimmune response, and in particular the development of T- and B-cells. One recent study on inflammatory proteins in MG revealed significantly increased serum levels of a proliferation-inducing ligand (APRIL), and cytokines IL-19, IL-20, IL-28A and IL-35 in MG as compared with controls^13^. Otherwise, no previous study has focused on defining a broad inflammatory circulating protein profile in MG patients and in different clinical MG subtypes.

Due to the lack of knowledge regarding the inflammatory circulating protein profile in MG, we analysed 92 different proteins associated with inflammation. Further, we analysed protein expressions within the clinical subgroups EOMG vs. LOMG, immunosuppressive medication, gender and thymectomy.

## Materials and Methods

### Subjects

Sera from 45 MG patients (24 women) were collected at the Neurology Clinics of Jönköping County Hospital and Uppsala University Hospital, Sweden. Sera from healthy controls (HC), matched for age and sex, were collected at Uppsala University Hospital transfusion unit, Sweden. Diagnostic criteria of MG included objective muscle fatigue and neurophysiological evidence of disturbed neuromuscular transmission (decrement on repetitive nerve stimulation and/or increased jitter on single fibre electromyography), further supported by detection of AChR antibodies^14^. Clinical classification of MG status according to the Myasthenia Gravis Foundation of America (MGFA)^15^ included only ocular weakness (MGFA class I) and generalized weakness of mild (MGFA class II), moderate (MGFA class III) or severe (MGFA class IV) degree predominantly affecting limb or axial muscles (subtype A) or bulbar muscles (subtype B). Additionally, the degree of clinical MG fatigue was assessed at the time for serum sampling with the MG Composite scale (MGC; range 0–50)^16^ All subjects gave their written informed consent to participate in the study. Ethical approval was granted by the Ethics Committee of the Ryhov County Hospital, Jönköping (Dnr 2014/459-31) and Uppsala University Hospital, Uppsala (Dnr 2010/446), Sweden and all experiments were performed in accordance with relevant guidelines and regulations.

### Protein analysis

The serum samples were analysed using the Olink Proseek Multiplex Inflammation I ^96×96^ kit (Olink Bioscience, Uppsala, Sweden), which analyses 92 human proteins related to inflammation and various inflammatory diseases (http://www.olink.com/proseek-multiplex/inflammation/). The analysis was performed at the Clinical Biomarkers Facility (Science for Life Laboratory, Uppsala, Sweden). The Proseek Multiplex analysis utilizes the Proximity Extension Assay (PEA) technology, as previously described^17^,^18^. In brief, antibodies labelled with oligonucleotide probes, bind in pairs to their specific target protein. These antibody pairs are linked to each other via their unique DNA oligonucleotide sequences, which hybridize when in proximity. The DNA sequences are then extended and amplified by quantitative real-time PCR. Details about data validation, limit of detection (LOD), specificity and reproducibility can be obtained via Olink’s website (http://www.olink.com/data-you-can-trust/validation/). Calibrator curves for correlating the normalized protein expression (NPX) values with actual concentrations are available at Olink’s website (http://www.olink.com/proseek-multiplex/inflammation/biomarkers/). A fold change value of at least 0.5 NPX is considered a biological difference, and not technical variation.

### Statistical analysis

The data was log base 2 transformed for the analysis in order to obtain data more similar to a normal distribution. 73 out of the 92 proteins followed a normal distribution, and for these proteins, an unpaired Welch t-test was performed for comparison of the two groups (MG versus HC). For comparison of the proteins that were not normally distributed, a Mann- Whitney U test was performed. The False discovery rate method was applied to manage multiple test errors. To investigate whether gender had any effect on the protein expression, a two-way ANOVA analysis was performed. The MG patients were divided into three sub-groups, depending on (1) early onset (<50 years of age) versus late onset (≥50 years or age) MG; (2) thymectomy versus no thymectomy and; (3) current treatment with immunosuppressant medication versus no immunosuppressive therapy. An unpaired two-tailed Welch t-test was performed to test different subgroup analysis of protein expression. A p-value < 0.05 was considered significant. A non-parametric dynamic principal component analysis (PCA) was performed to identify possible clusters of the proteins that differed the most between MG and HC.

## Results

### Clinical characteristics of MG patients

Three samples (2 HC and 1 patient) were excluded due to unacceptable technical variations and thus, the final cohort consisted of 87 subjects, 44 MG patients (23 women) and 43 healthy controls (23 women). In the MG patient cohort, mean age was 63 ± 17 years (range 27–87 years) and mean disease duration was 14 ± 12 years (range 1–48 years). The cohort included 22 EOMG and 22 LOMG patients and overall median MGFA disease severity classification was mild MG (MGFA class II; Table 1). 39 patients had verified AChR antibodies whereas the remaining 5 patients were seronegative for AChR and MuSK antibodies. 18 patients had undergone thymectomy and 16 patients had current treatment with immunosuppressive medication. The mean MGC score was 7.0 ± 7.1 (range 0–34) (Table 1).

### Elevated protein expressions in MG patients

**Figure 1:**
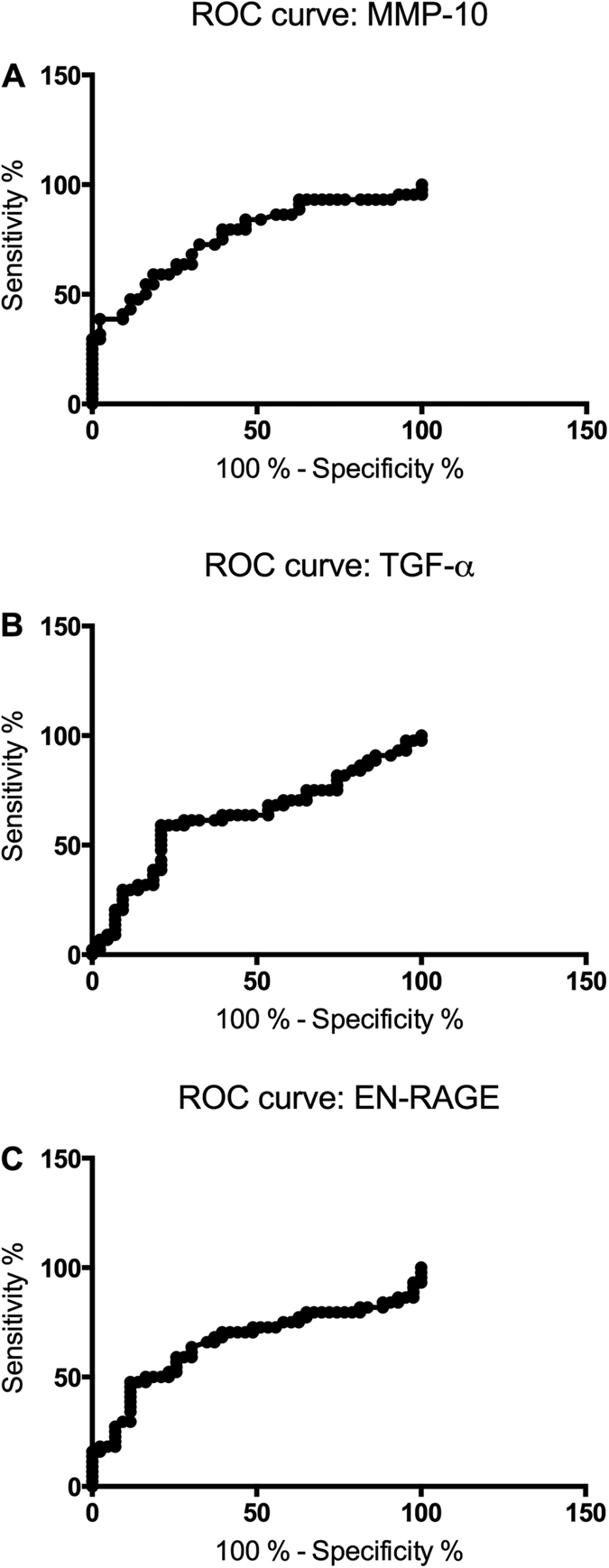
ROC curve of the three most significantly elevated proteins in sera of MG (myasthenia gravis) patients: MMP-10 (**A**), TGF-α (**B**) and EN-RAGE (**C**).

**Figure 2:**
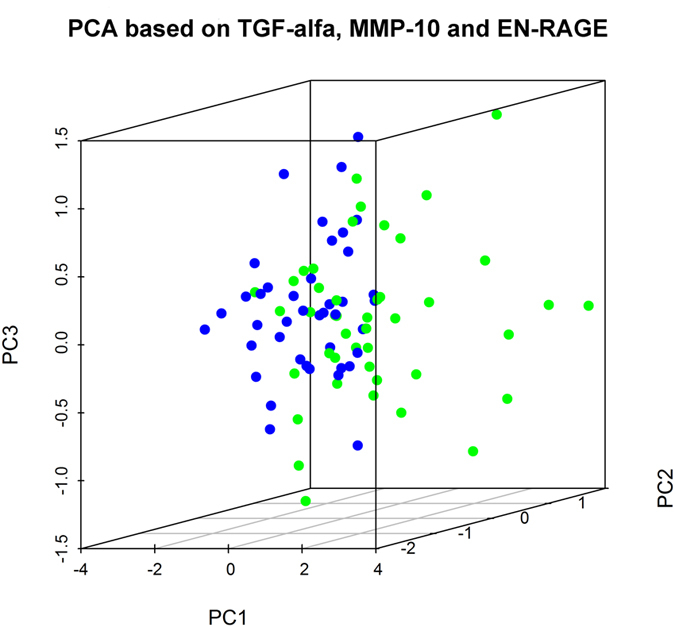
Dynamic three-dimensional principal component analysis (PCA) based on the three proteins with highest levels in MG patients: MMP-10, TGF-α and EN-RAGE. The PCA indicates a tendency for clustering in the MG patients. Blue, healthy controls (HC); green, myasthenia gravis (MG) patients.

The results from the assay are presented as normalized protein expression units (NPX), which are considered arbitrary units for each individual protein. Out of 92 assayed inflammatory proteins, 11 proteins were significantly elevated in the MG cohort (Table 2). The three proteins that were most significantly separated between MG patients and HC were matrix metalloproteinase 10 (MMP-10), transforming growth factor alpha (TGF-α) and extracellular newly identified receptor for advanced glycation end-products binding protein (EN-RAGE), all p < 0.01. The area under the curve (AUC) for these three inflammatory proteins was 0.76 for MMP-10 (Fig. 1A), 0.63 for TGF-α (Fig. 1B) and 0.66 for EN-RAGE (Fig. 1C). Thus, MMP-10 had the strongest association with MG. The PCA plot indicated tendency towards clustering in the HC group, although no absolute separation was present due to a greater dispersion in the MG patient group (Fig. 2). Other significantly increased levels of inflammatory proteins included nerve growth factor beta (β –NGF), cytokines IL-6, IL-8, IL-10 and IL-17A and C as well as chemokines C-C motif ligand 19 (CCL19) and C-X-C motif ligand 1 (CXCL1; Table 2). None of the investigated proteins had a lower expression in the MG group compared to HC group. Further, no significant correlation was found between any of the elevated proteins and clinical MGC score (Supplementary Table S1).

### Proteins associated with clinical MG subgroups

**Figure 3:**
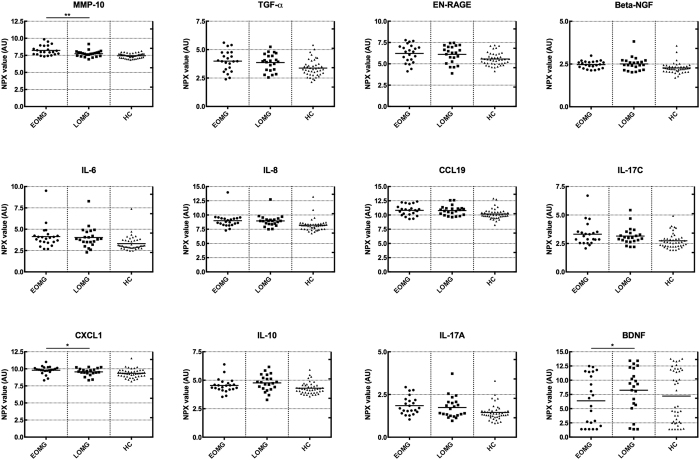
NPX levels in the different subgroups: EOMG, early onset myasthenia gravis; LOMG, late onset myasthenia gravis; HC, healthy controls; AU, arbitrary units. Lines indicate mean NPX value in each group. *p < 0.05, **p < 0.01. MMP-10, metalloproteinase 10; TGF-α, transforming growth factor alpha; EN-RAGE, extracellular newly identified receptor for advanced glycation end-products binding protein; beta-NGF, nerve growth factor beta; IL, interleukin; CCL19, C-C motif ligand 19; CXCL1, C-X-C motif ligand 1; BDNF, brain-derived neurotrophic factor.

In the subgroup two-way ANOVA analysis, none of the proteins were expressed differently in female compared to male MG patients. Patients in the EOMG subgroup had significantly higher levels of MMP-10 (p = 0.0092; Fig. 3) as well as CXCL1 (p = 0.033; Fig. 3) compared to the LOMG group. Brain-derived neurotrophic factor (BDNF), although not significantly separated in the MG group as a whole, was markedly elevated in LOMG compared to EOMG patients (p = 0.0318; Fig. 3). No proteins were clearly separated in the subgroups of immunosuppressive medication or previous thymectomy. Although not significant, some other proteins were separated with at least 0.5 NPX difference between group means (Table 3).

## Discussion

In this study, we found that 11 of the 92 different inflammatory proteins were elevated in the sera of MG patients. The fact that the dispersion of inflammatory proteins was greater in the MG group than the control group was largely expected, due to the heterogeneous nature of MG, with several subgroups and different clinical presentations. The protein that was most strongly associated with MG was MMP-10, also known as Stromelysin-2, which is a member of the metalloproteinase family. Metalloproteinases enzymatically break down the extracellular matrix, resulting in degradation of collagens, fibronectin and gelatines, both in physiological and disease processes. One previous study linked elevated serum levels of MMP-10 to sepsis in human patients^19^. Another study found that levels of MMP-10, secreted by microglia and macrophages, were considerably elevated in experimental autoimmune encephalomyelitis (EAE), which is an animal model for multiple sclerosis, and that these levels correlated with the severity of symptoms^20^. The model of EAE has many similarities to the animal model of experimental autoimmune MG (EAMG), for example in its response to immunomodulators^21^.

TGF-α is a growth factor that has important roles for epithelial proliferation and differentiation, mediated through its receptor the epidermal growth factor receptor (EGFR). TGF-α is mainly produced in macrophages, neurons, astrocytes and keratinocytes. Stimulation of proliferation via the EGFR signalling pathway results in inhibition of apoptosis and angiogenesis. Further, TGF-α has neurotrophic characteristics, such as stimulating proliferation and differentiation of neural precursor cells in the developing brain, and is able to stimulate proliferation in rat brain after stroke^22^. TGF-α also reduces the infarct size following stroke in mice, and stimulates angiogenesis and neurogenesis^23^. Although this neurotrophic role of TGF-α is known in the brain, nothing is so far known of its role in the peripheral nervous system.

EN-RAGE, also known as protein S100-A12, is a protein that binds to calcium, zinc and copper. Just like MMP-10 and TGF-α, EN-RAGE is involved in the cell cycle progression and differentiation and intriguingly this protein actually inhibits metalloproteinases. Additionally, EN-RAGE possesses pro-inflammatory properties, and is involved in inflammation by activating mast cells and monocytes. Further, EN-RAGE is induced by TNF-α in neutrophils and is upregulated by IL-6 in macrophages^24^. Previous studies proved that IL-6 is upregulated in thymic epithelial cells in MG patients^25^ and impaired expression of IL-6 among other cytokines is believed to play a role in the inflammatory response in MG^26^. EN-RAGE is a ligand for the receptor for advanced glycation end products (RAGE), a well-known pro-inflammatory receptor that is involved in diabetic neuropathy, cardiovascular disorders and neuroinflammatory and neurodegenerative disorders^27^,^28^. Binding of EN-RAGE to RAGE activates the transcription factor NF-κB, induces secretion of cytokines and has pro-inflammatory effects on lymphocytes, neutrophils and endothelial cells^29^.

β-NGF is another well-known protein, which belongs to the neurotrophin family. It is important for the survival and development of neurons in both the central nervous system (CNS) and the peripheral nervous system (PNS). However, it also has a role in the inflammatory response due to its release by T- and B-cells, mast cells, eosinophils and macrophages. β-NGF participates in a complex network of inflammation, being upregulated by inflammatory cytokines, activating inflammatory cells and stimulating further cytokine release as well as chemotaxis^30^.

Interleukins are well-known regulators of the inflammatory response in autoimmune diseases. Both IL-6 and IL-10 are produced by activated B cells (as well as T-cells), and affects macrophages as well as immunoregulatory T-cells^31^,^32^ and B-cells. IL-6 is particularly important for the development of the autoimmune response in EAMG^33^, by stimulating the development of Th17 cells. Th17 cells in turn secrete the cytokine IL-17A^32^, and a recent study found that plasma levels of IL-17A were increased in MG patients and that higher levels of IL-17A correlated with greater severity of the disease^34^. Furthermore, another study presented evidence that serum levels of IL-17 correlates with the AChR antibody titre^35^. IL-17C, another member of the IL-17 family, has not previously been linked to MG. Nevertheless, IL-17C is important in the development of EAE, and in regulating the autoimmune response through its receptor, IL-17E, which is highly expressed in Th17 cells^36^. Our data support the previous report on upregulation of the pro-inflammatory cytokine IL-8 in the sera of MG patients^37^. IL-8 is known to attract neutrophils and T-cells and has been linked to the occurrence of thymoma in MG patients^38^. In line with previous studies, our findings further validate the importance of these inflammatory cytokines in MG and emphasize the possibility that they could serve as targets for more specific novel MG treatments.

CCL19 is a cytokine that is secreted by medullary thymic epithelial cells and promotes migration of T-cells towards the medullary zone, as well as exporting mature thymocytes towards the periphery. CCL19, along with chemokine CCL21, is known to be upregulated in the thymus of MG patients with thymic hyperplasia^39^. Even though CCL21 was not included in our assay, our data support previous findings of the important role of CCL19 also in the circulation of MG patients. Another chemokine that was upregulated in the sera of MG patients is CXCL1, which stimulates chemotaxis of neutrophils and is produced by several cells in the immune system, such as macrophages and neutrophils^40^,^41^. Intriguingly, CXCL1 and IL-8 share the same receptor, CXCR2, which is present on mast cells, neutrophils, basophils and T-cells^42^. CXCR2 knockout mice present with defect neutrophil recruitment in wounds and delayed healing of wounds^43^. The fact that two ligands for CXCR2 are both elevated in the sera of MG patients reinforces the possibility that CXCR2 plays a crucial part in the autoimmune response in MG patients.

In the sub-group comparing onset of MG, patients with LOMG had almost four times higher levels of BDNF than the patients with EOMG. The ratio was almost the same when comparing patients who underwent thymectomy with those who did not. This was expected, since patients with LOMG are rarely treated with thymectomy unless they have a thymoma. Hence, the reason for the different BDNF levels is most likely due to the thymus differences between EOMG and LOMG, rather than effects from the thymectomy. Since BDNF is a neurotrophic protein, it is essential for differentiation and growth of neurons both in the CNS and the PNS. Notably, pro-inflammatory cytokines actually cause a reduction of BDNF gene expression^44^. One possible explanation for the higher levels of BDNF in patients with LOMG could be that their disease is less driven by inflammation compared to EOMG. The fact that EOMG patients more often have thymus hyperplasia and more effect of thymectomy is in conjunction with this finding. However, apart from neurons, inflammatory cells also produce BDNF, which may serve a neuroprotective role during inflammation^45^. Furthermore, BDNF also plays an important role in late-phase long-term potentiation and long-term memory storage^46^,^47^. Previous studies indicate that a fraction of MG patients do suffer spatial, logical and visual memory deficits, probably due to impaired cholinergic signaling in the CNS, since AChR antibodies are also found in the cerebrospinal fluid^48^. Impaired expression of BDNF could also be a possible reason for the cognitive deficits in some MG patients^49^.

Since the results from this protein panel are expressed in arbitrary units, the protein levels cannot be compared to other proteins, or even to the same protein in another assay due to usage of different batches of antibodies. This may be considered a drawback of the method, and thus limits the potential of this particular protein assay in its role to identify possible biomarkers for treatment response or inflammatory activity. The fact that many patients had current treatment with immunosuppressive treatment could also lower the levels of certain circulatory inflammatory proteins. Nevertheless, these findings provide valuable insight into the role of inflammatory mechanisms in MG pathophysiology.

In summary, we found eleven elevated inflammatory proteins in the sera of MG patients. These proteins, out of which MMP-10, TGF-α and EN-RAGE were the most significant, all have possible functions as new biomarkers of inflammatory activity, and possibly treatment response in MG.

## Additional Information

**How to cite this article**: Molin, C. J. *et al*. Profile of upregulated inflammatory proteins in sera of Myasthenia Gravis patients. *Sci. Rep.* **7**, 39716; doi: 10.1038/srep39716 (2017).

**Publisher's note:** Springer Nature remains neutral with regard to jurisdictional claims in published maps and institutional affiliations.

## Supplementary Material

Supplementary Table 1

## Figures and Tables

**Table 1:** Clinical data on the 45 MG patients included in the study.

Age/sex	Disease dur	EOMG/LOMG	Tx	IS therapy during disease course	IS now	AChR+	MGFA/MGC	Hist
27/F	3	EOMG	No	None	No	Yes	IIIA/8	NA
29/F	6	EOMG	Yes	Pred	Yes	Yes	IIIB/17	HP
30/F	2	EOMG	No	None	No	Yes	IIA/5	NA
33/F	17	EOMG	Yes	None	No	Yes	I/5	Missing
34/F	4	EOMG	Yes	Pred	Yes	Yes	IIB/0	HP
35/F	15	EOMG	Yes	None	No	Yes	IIA/0	HP
39/F	32	EOMG	Yes	None	No	Yes	IIB/0	HP
46/F	13	EOMG	No	None	No	Yes	I/4	NA
49/F	28	EOMG	Yes	Pred, AZA	Yes	Yes	IIB/10	NA
49/F	40	EOMG	Yes	AZA	No	No	IIA/4	Norm
52/F	11	EOMG	Yes	CyA	Yes	Yes	IIA/0	HP
53/F	19	EOMG	Yes	AZA	Yes	Yes	IIB/14	HP
57/F	34	EOMG	Yes	None	No	Yes	IIB/9	Missing
61/F	27	EOMG	Yes	None	No	Yes	IIA/14	HP
66/F	24	EOMG	No	None	No	Yes	IIIB/17	NA
67/F	19	EOMG	Yes	Pred	Yes	Yes	IIB/12	THY
68/F	48	EOMG	No	None	No	Yes	IIB/11	NA
69/F	41	EOMG	Yes	Pred, AZA	No	Yes	IIA/0	Missing
73/F	33	EOMG	No	Pred, IvIg	Yes	No	I/5	NA
84/F	2	LOMG	No	AZA	Yes	Yes	IIB/8	NA
69/F	11	LOMG	No	None	No	No	IIA/13	NA
76/F	1	LOMG	No	None	No	Yes	IIIA/13	NA
86/F	6	LOMG	No	None	No	Yes	IIB/2	NA
42/M	12	EOMG	No	None	No	No	I/1	NA
56/M	21	EOMG	Yes	Pred, AZA, CyA	Yes	Yes	IIB/11	HP
59/M	23	EOMG	Yes	Pred, CyA	Yes	Yes	IIB/6	THY
59/M	8	LOMG	No	Pred	Yes	Yes	IIB/0	NA
66/M	2	LOMG	No	Pred, AZA, IvIg	Yes	Yes	IIIB/15	NA
69/M	1	LOMG	No	None	No	Yes	IIIA/13	NA
69/M	6	LOMG	No	Pred, AZA	Yes	Yes	IIB/3	NA
71/M	8	LOMG	Yes	None	No	Yes	IIA/3	Norm
71/M	16	LOMG	Yes	None	No	Yes	IIB/5	THY
73/M	1	LOMG	No	None	No	Yes	IIB/3	NA
74/M	15	LOMG	No	None	No	Yes	0/0	NA
75/M	6	LOMG	No	Pred, AZA	Yes	Yes	0/0	NA
76/M	1	LOMG	No	None	No	Yes	IIA/2	NA
78/M	2	LOMG	No	AZA	Yes	Yes	IIIB/18	NA
78/M	10	LOMG	Yes	Pred, AZA	Yes	Yes	0/0	Missing
79/M	13	LOMG	No	Pred	No	Yes	IIA/2	NA
79/M	19	LOMG	No	None	No	No	0/0	NA
80/M	2	LOMG	No	None	No	Yes	IIB/15	NA
86/M	10	LOMG	No	AZA	No	Yes	IIA/2	NA
87/M	1	LOMG	No	None	No	Yes	IVB/34	NA
87/M	15	LOMG	No	None	No	Yes	IIA/3	NA

Onset, age at onset of MG; Disease dur, duration in years from MG diagnosis; F, female; M, male; Disease dur, years from MG diagnosis; Tx, thymectomy; IS, immunosuppressive medication; pred, prednisone; AZA, azathioprine; IvIg, intravenous immunoglobulin; CyA, cyclosporine; MGFA, Myasthenia Gravis Foundation of America where 0, remission; I, ocular weakness, II, mild weakness, III, moderate weakness and IV severe weakness. MGC, myasthenia gravis composite score; Hist, thymus histology; NA, not applicable; Norm, normal; HP, hyperplasia; THY, thymoma.

**Table 2:** Significantly increased levels of inflammatory proteins between MG patients and healthy controls.

Protein	*P*-value
MMP-10	0.0004
TGF-α	0.0017
EN-RAGE (Protein S100-A12)	0.0054
β -NGF	0.0133
IL-6	0.0220
IL-8	0.0220
CCL19	0.0220
IL-17C	0.0256
CXCL1	0.0256
IL-10	0.0256
IL-17A	0.0348

MMP-10, metalloproteinase 10; TGF-α, transforming growth factor alpha; EN-RAGE, extracellular newly identified receptor for advanced glycation end-products binding protein; β -NGF, nerve growth factor beta; IL, interleukin; CCL19, C-C motif ligand 19; CXCL1, C-X-C motif ligand 1.

**Table 3:** Separated proteins (> 0.5 NPX fold change of log2 data) between the different subgroups.

Increased NPX for patients w/o Tx	Tx	No Tx	Fold change
BDNF	6.3	8.0	1.7
CXCL9	8.1	8.9	0.8
**Increased NPX for patients w/o IS**	**Current**	**None**	**Fold change**
CXCL11	8.3	9.1	0.7
FGF-19	9.0	9.6	0.7
4E-BP1	5.9	6.6	0.6
TNFSF14	4.4	5.0	0.6
SIRT2	4.4	4.9	0.6
CCL4	7.2	7.8	0.6

BDNF, brain-derived neurotrophic factor; CXCL9, C-X-C motif chemokine 9; CXCL11, C-X-C motif chemokine 11; FGF-19, Fibroblast growth factor 19; 4E-BP1, Eukaryotic translation initiation factor; TNFSF14, 4E-binding protein 1; SIRT2, SIR2-like protein 2; CCL4, C-C motif chemokine 4. Tx, thymectomy; IS, immunosuppressive medication.
