# Supplementary material for: Profile of upregulated inflammatory proteins in sera of Myasthenia Gravis patients
Source: Sci Rep. 2017 Jan 3;7:39716. doi: 10.1038/srep39716 (PMC5206650; doi:10.1038/srep39716)
Supplement: Supplementary Table 1 [file srep39716-s1.pdf]

Profile of upregulated inflammatory proteins in sera of Myasthenia Gravis patients

Carl Johan Molin[+], Elisabet Westerberg[+], Anna Rostedt Punga*

Uppsala University, Department of Neuroscience, Clinical Neurophysiology, BMC, Husargatan 3, 75237 Uppsala, Sweden

[+] these authors contributed equally to this work

*** Corresponding author:** anna.rostedt.punga@neuro.uu.se

Supplementary table 1.

**Protein**	***r***	***P*-value**
Matrix metalloproteinase 10 (MMP-10)	-0.13	0.38
Transforming growth factor alpha (TGF-α)	-0.03	0.86
Protein S100-A12 (EN-RAGE)	-0.04	0.80
Beta-NGF	-0.11	0.50
IL-6	-0.24	0.12
IL-8	-0.02	0.92
CCL19	0.11	0.48
IL-17C	-0.11	0.48
CXCL1	0.10	0.52
IL-10	-0.07	0.67
IL-17A	-0.08	0.62

Correlation analysis between MGC score and NPX value of each protein. *r* = Pearson correlation coefficient.
